# The Effect of *Dobbs v. Jackson Women’s Health Organization* on Clinical Diagnosis of Postpartum Depression

**DOI:** 10.36469/001c.129633

**Published:** 2025-02-26

**Authors:** Onur Baser, Yuanqing Lu, Facundo Sepulveda, Ariani Alemzadeh, Amy Endrizal

**Affiliations:** 1 Department of Economics Boğaziçi University, Istanbul, Turkiye; 2 School of Public Health City University of New York, New York; 3 Columbia Data Analytics, Ann Arbor, Michigan; 4 Department of Economics University of Santiago, Chile; 5 School of Public Health City University of New York

**Keywords:** postpartum depression, women’s health, abortion, maternal health risks, maternal complications, Medicaid, anti-abortion laws

## Abstract

**Background:** The 2022 US Supreme Court decision in *Dobbs v. Jackson Women’s Health Organization* eliminated the constitutional right to abortion and activated trigger laws in 21 states, either banning or significantly restricting abortion access. This study estimated changes in postpartum depression (PPD) diagnoses after *Dobbs* in states with trigger laws vs those without. **Methods:** Medicaid data from Kythera Labs spanning December 2019 to June 2024 were utilized. Difference-in-difference models assessed changes in PPD diagnosis rates post-*Dobbs* (21 trigger states, 29 non-trigger states). **Results:** Women in trigger states were younger (mean, 26.53 vs 27.98 years), more likely to reside in low socioeconomic status areas (41.28% vs 24.42%) and less likely to have obstetrical complications (66.06% vs 77.36%), maternal complications (16.41% vs 18.9%), and lifestyle risk factors (13.58% vs 21.17%). Baseline PPD diagnosis rates were 8.51% in trigger states and 12.66% in non-trigger states. Post-*Dobbs*, PPD diagnosis rates were 10.20% in trigger states and 14.34% in non-trigger states. **Conclusions:** Overall, women in states with abortion trigger laws experienced a small positive but statistically insignificant increase in PPD diagnoses following *Dobbs* compared with those in non-trigger states.

## INTRODUCTION

The US Supreme Court’s 1973 ruling in *Roe v Wade* rendered the authority to regulate and pass abortion-related healthcare law to the federal government.^1^ On June 24, 2022, the Supreme Court overturned that ruling with its decision in *Dobbs v Jackson Women’s Health Organization*, 597 US 215.^2^ The *Dobbs* decision triggered significant changes in abortion laws across the United States, with 21 states poised to ban or severely restrict abortion access. Specifically, 14 states—Alabama, Arkansas, Idaho, Indiana, Kentucky, Louisiana, Mississippi, Missouri, North Dakota, Oklahoma, South Dakota, Tennessee, Texas, and West Virginia—have implemented near-total or total bans on abortion.^3^ Additionally, 7 states—Arizona, Florida, Georgia, Nebraska, North Carolina, South Carolina, and Utah—have established gestational limits on abortion, ranging from 6 to 18 weeks.^3^

Abortion is the termination of a pregnancy, which can be performed through two primary methods: medication abortion, involving the use of drugs to end the pregnancy, and surgical abortion, a procedure that physically removes the pregnancy from the uterus. Arguments about the legal status of abortion generally focus on ethical, moral, and religious issues. However, one aspect of the discourse surrounding restricted abortion legislation centers on the detrimental impacts on mental well-being. Supporters of abortion rights contend that the implementation of stringent abortion legislation at the state level amplifies the psychological distress experienced by women when confronted with a pregnancy that poses significant health and financial risks.^4^ Opponents of abortion rights contend that the implementation of stringent state abortion laws can assist women in mitigating their mental pain or psychological discomfort associated with their pregnancies by preventing impulsive and irrevocable decisions that may lead to subsequent remorse.^5^ In fact, the issue of potential adverse mental health effects following abortion has been invoked by policy makers as justification for enacting more restrictive abortion legislation. This argument gained prominence in the US Supreme Court’s decision in *Gonzales v. Carhart*,^6^ which upheld the Partial-Birth Abortion Ban Act of 2003. Writing for the majority, Justice Anthony Kennedy stated, “While we find no reliable data to measure the phenomenon, it seems unexceptionable to conclude some women come to regret their choice to abort the infant life they once created and sustained. Severe depression and loss of esteem can follow.”^7^ Hence, the impact of restrictive state abortion laws on the occurrence of PPD is a matter that should be investigated empirically.

The mental health consequences of induced abortion have been a subject of extensive research and debate in the medical and social sciences.^8–10^ Early studies in this field often focused on the “abortion trauma syndrome” hypothesis, which posited that women may suffer adverse psychiatric effects, including depression, following an induced abortion.^9,11^ However, subsequent research has highlighted significant methodological challenges in assessing these effects, particularly in the context of universal abortion access.^11^ The challenge of identifying an appropriate control group has been a central issue in this research area. Women who seek abortions after gestational limits or carry unwanted pregnancies to term may differ systematically from those who obtain abortions within legal timeframes, potentially confoundingresults.^12–14^

*Dobbs* allowed states to restrict abortion rights, providing a unique natural experiment to assess the effects of abortion access on mental health outcomes.^2^ As state decisions to restrict abortion rights can be considered largely independent of unobserved factors affecting abortion demand, comparing states with restricted vs unrestricted access offers a novel approach to identify the effects of abortion access. This paper aims to contribute to this evolving body of literature by examining the mental health impacts of restricted abortion access in the post-*Dobbs* landscape. By leveraging the variation in state-level abortion policies, we seek to provide robust evidence on the relationship between abortion access and mental health outcomes, addressing long-standing methodological challenges in this field of study.

The findings reveal notable differences in baseline characteristics between trigger and non-trigger states. Pre-*Dobbs* PPD diagnosis rates were 8.51% in trigger states and 12.66% in non-trigger states. Post-*Dobbs*, these rates increased to 10.20% in trigger states and 14.34% in non-trigger states. While the study found a small, statistically non-significant increase in PPD diagnosis rates in trigger states post-*Dobbs*, this may translate to numerous PPD cases annually nationwide. This trend warrants attention due to potential long-term impacts on maternal-infant bonding and child development.

## METHODS

### Study Design and Data Source

We conducted a retrospective cohort study utilizing Kythera Medicaid files from January 2019 to June 2024. The Kythera database is a comprehensive healthcare claims repository that encompasses medical and pharmacy claims data, providing coverage for approximately 79% of the US patient population.^15^ This extensive database integrates claims from various sources, including commercial, Medicare, and Medicaid programs.^16^ The data set contains 60% of the Medicaid population across all 50 states and includes de-identified patient numbers, age, gender, insurance types (fee-for-service vs managed care), zip codes, diagnoses according to the *International Classification of Diseases* (ICD-10), Current Procedural Terminology (CPT) codes, and National Drug Codes (NDC) for medications.^15^ Each patient is assigned a unique identifier, which links all encounters and facilitates longitudinal analysis.

Kythera’s data acquisition process encompasses the comprehensive claim adjudication cycle. When a claim is submitted to a payer, Kythera’s system captures the iterative communication between the healthcare provider and the payer, as mediated by the claim adjudicator—typically a third-party technology vendor facilitating this exchange. This approach allows for a detailed record of claim progression through various stages of review and negotiation. Additionally, Kythera augments its data collection by incorporating a subset of transactions from revenue cycle management systems, which often function as point-of-sale interfaces in healthcare settings. Furthermore, a portion of data is sourced directly from healthcare providers, offering a more immediate and unfiltered perspective on claim initiation. Reference data, including diagnostic codes, procedural classifications, and pharmaceutical information, are obtained from authoritative industry sources such as the Food and Drug Administration and American Medical Association. Directories encompassing provider and payer information are constructed internally through a synthesis of public and proprietary sources, complemented by the integration of metadata derived from transactional records. The comprehensive details of the Kythera data have been documented in previous publications,^17,18^ and the healthcare outcomes derived from these data have been validated and compared with other data sets for consistency and accuracy.^19^ In particular, the study compared healthcare utilization patterns across 18 different diseases using data from 3 sources: 2 established closed claims data sets (MarketScan® and PharMetrics® Plus) and 1 open claims data set.^15^ For each disease, the researchers analyzed various healthcare utilization metrics, including inpatient stays, emergency department visits, outpatient use, and length of stay. The comparison revealed that the estimates derived from the Kythera data set were statistically similar to those from the closed claims data sets.^19^

### Exposure and Other Measures

**Figure 1.**
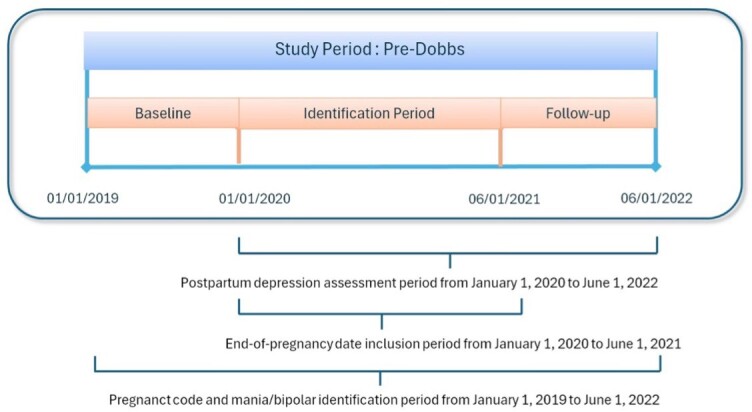
Pre-*Dobbs* Study Period Timeline Visualization

**Figure 2.**
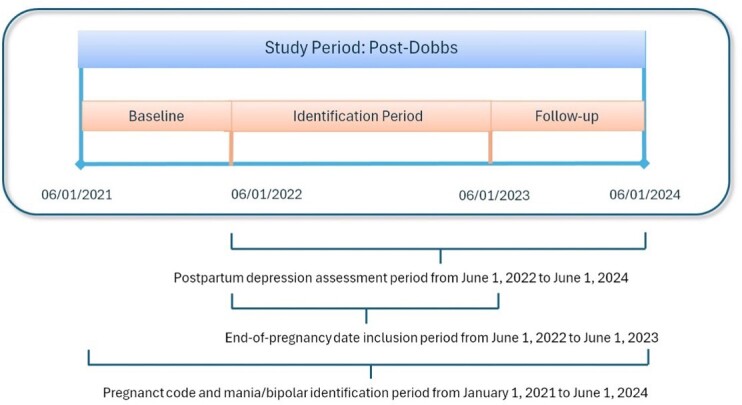
Post-*Dobbs* Study Period Timeline Visualization

Two distinct analytic samples were created, corresponding to the periods before and after the June 2022 *Dobbs* decision. The study population comprised women aged 12 to 55 years who experienced a pregnancy resulting in a live birth or stillbirth between January 2019 and June 2024 (study period). The pregnancy date was designated as the index date. Women whose pregnancy concluded between January 2020 and June 2021 were assigned to the pre-*Dobbs* cohort, while those with pregnancy dates between June 2022 and June 2023 constituted the post-*Dobbs* cohort. To ensure comprehensive assessment of comorbidities, maternal, obstetrical, and lifestyle risk factors, a 12-month continuous enrollment period was required prior to the index date. Similarly, a 12-month continuous enrollment period post-index date was mandated to capture PPD diagnoses (**Figures 1 and 2**).

PPD was identified using the *International Classification of Diseases, Tenth Revision, Clinical Modification* (ICD-10-CM) code F53*. The diagnostic criteria included at least 1 inpatient claim, or 1 outpatient claim for PPD, or a minimum of 1 inpatient or 2 outpatient claims for major depressive disorder occurring at least 27 days apart within 12 months following the end-of-pregnancy date or during the third trimester of pregnancy. To isolate PPD as a distinct condition, women with a history of mania or bipolar disorder during the study period were excluded. Furthermore, to differentiate new-onset depressive illness in the postpartum period from ongoing episodes, patients with major depressive disorder prior to their last menstrual period were also excluded from the analysis **(Supplemental Figure S1).**

To define the exposure and comparison groups, we constructed a binary variable indicating residence in one of the 21 states with trigger laws or in any of the 29 non-trigger states across 2 distinct periods. Key demographic covariates included age, region, urbanicity (urban, suburban, rural), and socioeconomic status (SES) as measured by the area deprivation index. Clinical factors encompassed comorbidity indexes, specifically the Charlson Comorbidity Score,^20^ Elixhauser Index,^21^ and Chronic Disease Score,^22^ as well as obstetrical, maternal, and lifestyle risk factors identified by ICD-10 codes (**Supplemental Table S1**). We also incorporated statewide variables that could potentially influence PPD diagnosis rates. These included the unemployment rate,^23^ education score, Substance Abuse and Mental Health Services Administration (SAMHSA) block grants for mental illness and substance use disorder prevention and treatment per capita,^24^ and the number of behavioral health care providers per 1000 state residents obtained from the US Census Bureau County Business Patterns.^25^ Additionally, we accounted for the presence of Medicaid Health Home programs and Affordable Care Act–related Medicaid expansion in each state.^26,27^ The variables and their descriptions are presented in **Table 1**.

**Table 1.** Detailed Covariate Coding

**Variable**	**Description**
Postpartum depression	Diagnosed using ICD-10-CM code F53* or major depressive disorder criteria
Trigger states	21 states that banned or severely restricted abortion access after *Dobbs*
Non-trigger states	29 states without abortion bans or severe restrictions after *Dobbs*
Pre-*Dobbs* period	January 2020–June 2021
Post-*Dobbs* period	June 2022–June 2023
Age	Women aged 12-55 years
Region	Midwest, Northeast, South, West
Urbanicity	Urban, suburban, rural
Socioeconomic status	Measured by area deprivation index
Comorbidity indexes	Charlson Comorbidity Score, Elixhauser Index, Chronic Disease Score
Obstetrical complications	Identified by ICD-10 codes
Maternal comorbidities	Identified by ICD-10 codes
Lifestyle risk factors	Identified by ICD-10 codes
Unemployment rate	State-level variable
Education score	State-level variable
SAMHSA block grants	Per capita funding for mental illness and substance use disorder prevention/treatment
Behavioral health providers	No. per 1000 state residents
Medicaid Health Home Programs	Presence in state
Medicaid expansion	Under Affordable Care Act

### Statistical Analysis

Descriptive analysis was conducted for trigger and non-trigger states pre- and post-*Dobbs*. A difference-in-difference approach was used to estimate changes states post-*Dobbs* and pre-*Dobbs* among residents of trigger relative to those living in non-trigger states.^28^ Difference-in-differences methods are a widely utilized strategy for assessing the impact of policies or programs that are implemented at a specific point in time, such as the enactment of new legislation.^29,30^ This method evaluates the impact of policy interventions by comparing changes over time in a group affected by the intervention (ie, *Dobbs*) to changes in a group not affected by it. The method attributes the difference in differences to the policy’s effect, assuming that, in the absence of the intervention, both groups would have followed similar trends.^31^ This approach is particularly useful because it incorporates information on temporal trends from the comparison group, potentially providing unbiased effect estimates. Consequently, difference-in-difference methods are sometimes preferred over interrupted time-series designs, which do not necessarily include a comparison group.^32^

For each pregnant woman (*i*), the model can be described as

$$y_{it}^{j}=\alpha +\alpha_{1}d_{t}+\alpha^{1}d^{j}+\beta_{1}d_{t}^{j}+\beta_{2}X_{it}^{j}+\beta_{3}Z_{st}^{j}+\gamma_{s}+\theta_{t}+\epsilon_{it}^{j},$$

where PPD rate *y* is indexed *j* for the group (for example, trigger states as equal to 1 vs non-trigger states as equal to 0) and *d_t_* = 1 if post period, 0 otherwise, *d ^j^* = 1 if in a trigger state vs 0 otherwise, and $d_{t}^{j}$ if in a trigger state post-period and vs 0 otherwise, *X ^j^* is the vector of characteristics of pregnant women such as age, SES, and comorbidities, $Z_{st}^{j}$ is the vector of characteristics of state variables such as unemployment and SAMHSA block grants for mental illness, and *γ^s^* and *θ^t^* represents state and year fixed effects, respectively. Further, α_1_ captures aggregate factors that would cause changes in PPD rates even in the absence of policy change, and α_1_ captures possible differences between the trigger and non-trigger states prior to policy change. The coefficient β_1_ is the causal effect of the decision on PPD rates.

Difference-in-difference models rely on the parallel-trends assumption, which posits that, in the absence of *Dobbs*, trends in PPD would have progressed similarly in both trigger and non-trigger states. To test this counterfactual assumption (ie, what would have happened to individuals in our study if they had not been exposed to the *Dobbs* decision) we assessed PPD rates between the trigger and non-trigger states 2 the pre-*Dobbs* periods to support similar pre-*Dobbs* trends.

In difference-in-difference studies, 2 primary forms of selection bias are of concern: across groups and across time. Selection bias across groups arises when individuals in states affected by the policy differ systematically (eg, in demographic or clinical characteristics). To mitigate this bias, our regression models incorporate controls for these differences. Selection bias across time occurs when the composition of groups changes over the study period. To address this, we restricted our sample to individuals who maintained continuous enrollment throughout the study duration. Additionally, because observations within the same state may not be independent, we clustered standard errors by state to account for potential intrastate correlations.

To address the potential heterogeneous effects of *Dobbs*, we employed a multifaceted analytical approach. First, we conducted a subgroup analyses, focusing on younger mothers (age <25 years). A large-scale study of over 1.1 million mothers found that self-reported PPD symptoms were most prevalent (10%) among 18-to 24-year-olds. Second, we implemented the Callaway and Sant’Anna estimator, a method specifically designed to address heterogeneous treatment effects in difference-in-difference models.^33^

We conducted sensitivity analyses to account for the unique legislative context in Texas. Specifically, we estimated models that included all states, excluded Texas, and focused solely on Texas. This approach was necessitated by the implementation of Texas Senate Bill 8 (SB8) in September 2021, approximately 10 months prior to *Dobbs*. Texas SB8 effectively prohibited abortions beyond 6 weeks of gestation through the imposition of civil penalties, resulting in a significant 50% reduction in abortion rates within the state.^34^

## RESULTS

Our study sample comprised 105 668 individuals in the pre-*Dobbs* period, with 48 775 residing in trigger states and 56 339 in non-trigger states. The post-*Dobbs* period included 45 359 individuals, of whom 22 785 were from trigger states and 22 574 from non-trigger states. **Table 2** presents the demographic, socioeconomic, and clinical characteristics, as well as state-level factors, for both trigger and non-trigger states during the pre-and post-*Dobbs* periods.

**Table 2.** Descriptive Baseline Characteristics

**Patient Characteristics**	**Pre-*Dobbs*** **(N = 105 668)**			**Post-*Dobbs*** **(N = 45 359)**		
	**Trigger States (N = 48 775)**	**Non-⁠trigger States** **(N = 56 399)**	**Std. Diff.**	**Trigger States (N = 22 785)**	**Non-⁠trigger States (N = 22 574)**	**Std. Diff.**
Age (years), mean^35^	26.53 (5.69)	27.98 (5.84)	0.25	26.63 (5.77)	28.26 (5.90)	0.28
Age group (years), n (%)						
12-17	997 (2.04)	711 (1.26)	0.06	469 (2.06)	254 (1.13)	0.08
18+	47 778 (97.96)	55 688 (98.74)	0.06	22 316 (97.94)	22 320 (98.87)	0.08
18-19	3344 (6.86)	2452 (4.35)	0.11	1528 (6.71)	957 (4.24)	0.11
20-24	15 687 (32.16)	14 361 (25.46)	0.15	7455 (32.72)	5447 (24.13)	0.19
25-29	14 613 (29.96)	17 470 (30.98)	0.02	6476 (28.42)	6834 (30.27)	0.04
30-34	9265 (19.00)	13 078 (23.19)	0.10	4471 (19.62)	5478 (24.27)	0.11
35+	4869 (9.98)	8327 (14.76)	0.14	2386 (10.47)	3604 (15.97)	0.16
Pandemic Period Indicator, n (%)	39 704 (81.40)	46 582 (82.59)	0.03	0 (0.00)	0 (0.00)	0.00
Region						
Midwest	2929 (6.01)	17 206 (30.51)	0.65	798 (3.50)	5782 (25.61)	0.66
Northeast	0 (0.00)	13 378 (23.72)	0.76	0 (0.00)	7851 (34.78)	1.03
South	37 660 (77.21)	3408 (6.04)	2.13	17 875 (78.45)	2079 (9.21)	1.95
West	8186 (16.78)	22 394 (39.71)	0.51	4 112 (18.05)	6862 (30.40)	0.29
Comorbidity score^35^						
Charlson Comorbidity Index score ≥1	1.11 (0.3807)	1.11 (0.3919)	0.02	1.12 (0.4142)	1.12 (0.3846)	0.00
Chronic Disease Score	2.11 (1.4193)	2.15 (1.4510)	0.02	2.33 (1.5012	2.19 (1.4785	0.09
Elixhauser Index score ≥1	1.60 (0.9928	1.71 (1.0775)	0.11	1.68 (1.0831)	1.83 (1.1755)	0.13
Location, n (%)						
Urban	5778 (11.85)	4642 (8.23)	0.12	2761 (12.12)	2028 (8.98)	0.10
Suburban	32 033 (65.68)	41 423 (73.45)	0.17	14 993 (65.80)	16 692 (73.94)	0.18
Rural	10 866 (22.28)	10 318 (18.29)	0.10	4963 (21.78)	3847 (17.04)	0.12
SES score, n (%)						
Low	20 136 (41.28)	13 771 (24.42)	0.37	9309 (40.86)	5303 (23.49)	0.38
Middle	15 611 (32.01)	18 243 (32.35)	0.01	7168 (31.46)	7443 (32.97)	0.03
High	11 558 (23.70)	23 278 (41.27)	0.38	5650 (24.80)	9389 (41.59)	0.36
ADI score, n (%)						
Low	25 071 (51.40)	17 315 (30.70)	0.43	11 650 (51.13)	7161 (31.72)	0.40
Middle	18 134 (37.18)	19 235 (34.11)	0.06	8507 (37.34)	8202 (36.33)	0.02
High	4881 (10.01)	19 532 (34.63)	0.61	2229 (9.78)	7092 (31.42)	0.55
Unemployment rate, n (%)						
Low	15 523 (31.83)	10 284 (18.23)	0.32	7491 (32.88)	4346 (19.25)	0.32
Middle	13 323 (27.32)	15 738 (27.90)	0.01	5807 (25.49)	8293 (36.74)	0.24
High	19 929 (40.86)	30 364 (53.84)	0.25	9487 (41.64)	9935 (44.01)	0.04
Behavioral healthcare providers, n (%)						
Low	33 489 (68.66)	5186 (9.20)	1.57	15 396 (67.57)	2023 (8.96)	1.51
Middle	12 476 (25.58)	25 599 (45.39)	0.41	5743 (25.21)	11978 (53.06)	0.59
High	2 810 (5.76)	25 601 (45.39)	0.99	1646 (7.22)	8573 (37.98)	0.79
SAMHSA grant, n (%)						
Low	12 229 (25.07)	7585 (13.45)	0.30	6196 (27.19)	3239 (14.35)	0.32
Middle	8546 (17.52)	20 641 (36.60)	0.43	4104 (18.01)	10 208 (45.22)	0.61
High	28 000 (57.41)	28 160 (49.93)	0.16	12 485 (54.79)	9127 (40.43)	0.29
Education score, n (%)						
Low	32 978 (67.61)	6211 (11.01)	1.45	15 247 (66.92)	2393 (10.60)	1.42
Middle	15 108 (30.97)	28 799 (51.06)	0.41	7178 (31.50)	11 538 (51.11)	0.40
High	689 (1.41)	21 376 (37.90)	0.99	360 (1.58)	8643 (38.29)	1.03
Medicaid Health Home for mental illness, n (%)	3824 (9.45)	18 880 (33.48)	0.59	1269 (6.65)	7264 (32.18)	0.67
ACA Medicaid expansion, n (%)	24 571 (50.38)	53 993 (95.73)	1.18	11 674 (51.24)	21 398 (94.79)	1.11
Any obstetrical complications,^a^ n (%)	32 219 (66.06)	43 628 (77.36)	0.25	15 832 (69.48)	18 150 (80.40)	0.25
Any maternal comorbidity, n (%)	8006 (16.41)	11 053 (19.60)	0.08	4321 (18.96)	4750 (21.04)	0.05
Any lifestyle risk factors, n (%)	6626 (13.58)	11 942 (21.17)	0.20	4024 (17.66)	5768 (25.55)	0.19
PPD diagnosis among pregnant women, n (%)	4149 (8.51)	7138 (12.66)	0.04	2325 (10.20)	3246 (14.34)	0.13

Abbreviations: ADI, area deprivation index; SES, socioeconomic status; Std.Diff., standardized difference.

^a^Detailed obstetrical, maternal complications and lifestyle factors can be found in **Supplemental Table S5.**

Analysis of demographic and clinical characteristics revealed notable differences between individuals in trigger and non-trigger states. Residents of trigger states were, on average, younger and more likely to reside in the South and in rural areas, characterized by lower SES and higher neighborhood deprivation scores. Interestingly, these individuals exhibited lower rates of maternal comorbidities and lifestyle risk factors and were less likely to experience obstetrical complications. These disparities in demographic, geographic, and clinical profiles between the two groups underscore the importance of these factors in interpreting the impact of *Dobbs* on maternal health outcomes (**Table 2**).

Compared with non-trigger states, patients residing in trigger states were more likely to live in areas characterized by higher unemployment rates and lower educational attainment. Additionally, these trigger states exhibited lower ratios of behavioral health care providers per 1000 residents, reduced per capita funding from SAMHSA block grants for mental illness and substance use disorder prevention and treatment, and were less likely to have implemented Medicaid Home Health Home programs or expanded Medicaid under the Affordable Care Act (**Table 2**).

**Figure 3.**
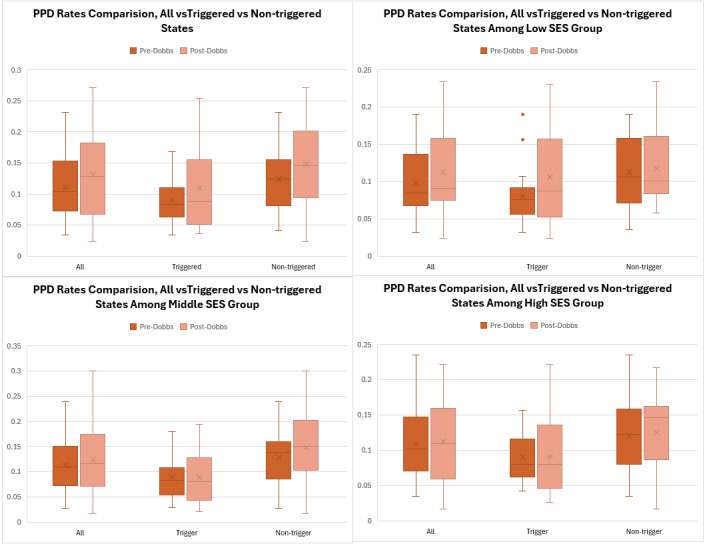
Variation of PPD Diagnosis Rates Among Trigger and Non-Trigger States Abbreviations: PPD, postpartum depression; SES, socioeconomic status.

Variation of PPD rates in trigger states between the pre-and post-*Dobbs* periods increased more than the variation of PPD rates in non-trigger states **(Figure 3**). The rate change for each state is presented in **Supplemental Figure S2**.

**Figure 4.**
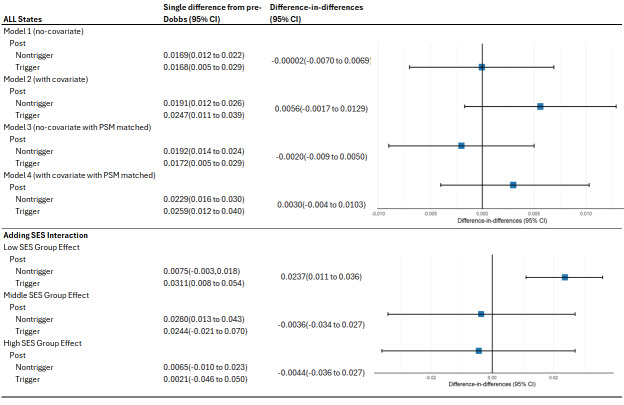
Difference-in-Difference Models Comparing PPD Diagnosis Rates Before and After *Dobbs* in Trigger vs Non-trigger States Abbreviations: CI, confidence interval; PSM, propensity score matching; SES, socioeconomic status. *All hypothesis testing was 2-sided. The 95% confidence intervals were estimated using cluster-robust standard errors, reported in parentheses.

PPD diagnosis rates for trigger and non-trigger states before *Dobbs* are shown in **Supplemental Figure S3**.) A statistical test of the parallel trends is provided in **Supplemental Table S2** (*P* = .687). Difference-in-difference analysis results are presented in **Figure 4**. The pre-*Dobbs* PPD diagnosis rates served as the reference for all models. We employed 4 regression approaches to analyze the data. First, we conducted an unadjusted analysis. Second, to mitigate selection bias, we adjusted for most of the covariates listed in **Table 2**. Any obstetrical complications, maternal comorbidities, and lifestyle risk factors were not included as covariates to avoid overcontrolling the model. We implemented propensity score–adjusted difference-in-difference models to further address potential bias.^36^ Third, regression was used to present the results without covariates; fourth, regression was used to present the results with covariates after propensity score adjustment. The single-difference coefficients represent the change in PPD diagnosis rates post-*Dobbs* in trigger states relative to the change in non-trigger states. All 4 models show statistically non-significant difference-in-difference estimates, as indicated by the confidence intervals crossing zero. The point estimates range from a slight decrease (-0.0020 in Model 3) to a small increase (0.0056 in Model 2) in PPD diagnosis rates in trigger states relative to non-trigger states. The most comprehensive model (Model 4), which includes covariates and propensity score matching, suggests a small increase of 0.30 percentage points in PPD diagnosis rates in trigger states compared with non-trigger states after *Dobbs*. However, this estimate is not statistically significant. The narrow confidence intervals across all models indicate relatively precise estimates, suggesting that if there is an effect of *Dobbs* on PPD diagnosis rates, it is likely to be small. The consistency of results across different model specifications (with and without covariates, with and without propensity score matching) strengthens the overall conclusion of no significant effect.

To address potential heterogeneity in treatment effects, subgroup analyses were stratified by maternal age (<25 years and ≥25 years) (**Supplemental Figure S4**) and a Callaway and Sant’Anna estimator, which is robust to treatment effect heterogeneity in staggered difference-in-difference designs. Stratification yielded statistically non-significant results (**Supplemental Table S3**). These findings suggest that the observed overall effects may not be driven by specific age groups and are robust to potential variations in treatment timing and intensity across subpopulations.

To ensure the reliability of our findings, we conducted a comprehensive sensitivity analysis. This analysis involved 2 key steps: First, we excluded Texas from the sample and re-ran the regressions, yielding results similar to the overall sample trends, as shown in **Figure 4**. Second, we conducted separate regression analyses using only data from Texas, which revealed that PPD diagnosis rates were significantly higher in Texas following *Dobbs* (**Supplemental Table S4**). We also conducted sensitivity analyses using different age ranges, including 18 to 45 years. The results of these analyses were also consistent with our primary findings, supporting our estimates across various age specifications.

## DISCUSSION

Our findings revealed no statistically significant difference in PPD diagnosis rates between trigger and non-trigger states following *Dobbs*. Several factors may explain the lack of the hypothesized “abortion trauma syndrome” effect in this context. Although the distance to the nearest abortion facility increased from an average of 43 miles one way before *Dobbs* to 330 miles at present, prior research indicates that individuals seeking abortions often travel to states where the procedure is legal.^37–39^ One estimate shows that under the current access landscape, approximately 75% of pregnant residents seeking access from banned states will travel to facilities in states without such bans.^37^ This is supported by current reports indicating increased abortion rates in states with legal abortion adjacent to states with bans.^40^

Income and resources of those women with financial means and support systems to travel out of state to obtain an abortion is a consideration in sample selection. Positive risk factors for PPD include psychiatric history, which might be assessed through diagnostic records, but also adverse life events, and social support, which may be harder to assess.^41^ Wealthier women may have a stronger social support system, which could prevent the onset of depression, whether due to the financial and logistical stress of travel to obtain the abortion or from the abortion itself.

Nevertheless, while we cannot observe who will choose to cross state lines to obtain an abortion, the evidence indicates that the women in our sample were very unlikely to migrate. Axelson and colleagues examined the characteristics of women who travel across state lines to have an abortion before *Dobbs* and found that the incidence for Medicaid patients is half that for the entire sample (3% vs 6% of abortion patients).^42^ This is likely because Medicaid patients tend to have lower incomes, and Medicaid generally only covers in-state care in most states. While selection bias cannot be entirely ruled out, we believe our sample minimizes its impact.

Rise in demand for programs like Aid Access, a nonprofit organization that provides mail-order access to medication abortion via overseas pharmacies, confirms that individuals unable to manage the logistics and costs for traveling for the service, undergo self-managed abortions without significant health risk.^43^ Moreover, enhanced access to a comprehensive range of contraceptive methods, including long-acting reversible contraceptives, may contribute toward a reduction in unintended pregnancies.^44^

This study adds to a small but growing literature on the public health effects of *Dobbs*. A number of contributions document increased demand for vasectomy procedures as an immediate consequence of the ruling.^35^ Other investigators have reported an increase in requests for telemedicine services that provide self-managed medication abortions.^43^ Of particular concern are 2 studies providing evidence for increased infant mortality following abortion bans. The first, by Gemmill et al,^45^ examines the effects on infant deaths of Texas’s SB8, which bans abortions after embryonic cardiac activity. The authors report a 12.7% increase in infant deaths in Texas over comparison states. The second study, by Singh et al,^46^ finds higher than expected nationwide infant death rates after *Dobbs*.

The impact of restrictive abortion policies has been studied in other countries, notably Mexico.^47^ The Mexican study primarily examined maternal morbidity, especially abortion-related morbidity and hemorrhage in early pregnancy. Similar to our study results, the Mexican study found small, generally nonsignificant effects from increasing sanctions on illegal abortion.

While our analysis revealed a small and statistically non-significant increase in PPD diagnosis rates of approximately 0.034% (model 4 without Texas), its potential impact on public health and individual well-being should not be overlooked. When applied to the approximately 4 million births occurring annually in the United States, this marginal increase may translate to an estimate as large as 1400 additional cases of PPD nationwide. Each case of PPD represents significant distress and impairment for the affected mother, with potential negative impacts on infant development and family functioning. The long-term consequences of PPD on maternal-infant bonding, child development, and family dynamics are well-documented, suggesting that even a small uptick in cases could have far-reaching effects persisting for years. Furthermore, this slight increase may signal the onset of a concerning trend that could accelerate if left unaddressed. Early identification of such trends, even when statistically non-significant, is crucial for implementing targeted interventions and prevention strategies before the issue potentially escalates. The recent US postpartum hospitalization study finds a 17% reduction in hospitalizations within 60 days postpartum associated with Medicaid expansion.^48^

Exacerbating this impact is growing evidence that states with restricted access to abortion will experience relative physician shortages, potentially including mental health specialists. The Association of American Medical Colleges reports a 5.2% relative decrease in obstetrics/gynecology residency applications in states with abortion bans compared with those without.^49^ Similarly, Tobin-Tyler et al report that obstetrics/gynecology specialists find themselves left in a difficult legal position when making treatment decisions in restricted-abortion states.^50^ In the meantime, further ongoing litigation leaves physicians’ ability to practice within the standard of care uncertain.^50^

As *Dobbs* has the potential to modify the entire landscape of access to health care for women in states that restrict abortion, our results can best be interpreted as a snapshot just following the policy changes. It is likely that women’s mental health will be further placed under stress by the reduction in health care options due to the detrimental effects of *Dobbs* on physician incentives.

### Limitations

Our findings may have limited generalizability due to the exclusion of commercial health insurance deliveries, which comprise approximately 60% of all deliveries in the United States. This exclusion is particularly important given that SES, a significant factor for PPD rate of Medicaid enrollees, tends to be systematically lower than that of privately insured individuals. This study also does not include a discussion on race—another important factor for PPD rate—due to the absence of a race variable in the Kythera data set.

Our estimates demonstrating the association between the policy and the outcome are presented after controlling for observable factors such as age, comorbidities, SES, and statewide differences that may affect PPD risk. However, there may be unobservable factors that could confound the estimates, including genetics, social support, or life events. To the extent that these unobservable factors are correlated with the policy variable, our study may introduce bias in estimating the relationship between the *Dobbs* decision and PPD risk.

Another limitation of our data set is the inability to identify patients who have traveled from states with trigger laws to those without. Consequently, the forced births remaining in our sample are likely to represent a subgroup of women with exceptionally limited resources to support either abortion-related travel or access to medication abortion. While this situation could potentially introduce bias into our analysis, it is important to note that our data set comprises Medicaid beneficiaries, representing a relatively homogeneous population in terms of SES. As such, we posit that any bias stemming from socioeconomic differences or the ability to travel to nearby states for abortion services is likely to be minimal. We have also conducted a sensitivity analysis to address potential bias arising from patients traveling to another state for care. Specifically, we tested whether our results remain consistent when patients from trigger states that border non-trigger states are excluded. For this analysis, we focused on Alabama and Georgia, as these are the only states with no borders to non-trigger states. The findings from this sensitivity analysis were consistent with the original results of our study, thus reinforcing the robustness of our conclusions.

Administrative data sets are valuable resources for research, but they come with several important limitations. Administrative data often contain inaccurate coding, missing information, and inconsistencies across sources.^51^ This stems from the fact that these data sets are typically collected for billing or operational purposes rather than research.^52^ Coding practices can vary between institutions and over time, leading to potential misclassification of diagnoses or procedures.^53^ The scope of administrative data is generally limited to information relevant for administrative purposes. They often lack detailed clinical data, patient-reported outcomes, or important contextual factors.^54^ This can result in unmeasured confounding when used for research. However, we were able to control for several important patient risk factors, including age, several comorbidity indexes, and individual comorbidities specific to PPD. Our analysis focused on PPD diagnoses rather than PPD itself due to the limitations of the administrative data. It is well-documented that PPD is notoriously underdiagnosed, and the literature suggests that this underdiagnosis occurs along socioeconomic lines.^55,56^ Our findings pertain to diagnosed cases of PPD, acknowledging that the true prevalence of PPD may be higher. Therefore, changes in coding practices, policy shifts, or evolving clinical definitions can create artificial trends that may be mistaken for true changes in disease incidence or healthcare utilization.

## CONCLUSION

Our study contributes to the ongoing discourse on the mental health implications of abortion legislation by providing one of the first empirical analyses of PPD rates following *Dobbs*, utilizing a large-scale Medicaid data set spanning December 2019 to June 2024. By employing a robust difference-in-differences methodology and controlling for a comprehensive set of individual and state-level factors, while the study found no statistically significant increase in PPD diagnoses post-*Dobbs*, the small positive trend observed (approximately 0.034% increase) may translate to an estimated 1400 additional cases of PPD nationwide annually. Since each case of PPD represents significant distress for the affected mother and may have long-term consequences for maternal-infant bonding and child development, the potential impact on public health and individual well-being should not be overlooked, as the findings underscore the need for enhanced mental health support and screening in states with restricted abortion access. Our research also highlights potential disparities in healthcare access between trigger and non-trigger states. Policymakers should consider measures to improve access to mental health services, particularly in states with more restrictive abortion laws. This is especially crucial given the growing evidence of relative physician shortages in states with restricted abortion access, as noted by the Association of American Medical Colleges’ report on decreased obstetrics/gynecology residency applications in these states. In conclusion, while the immediate impact of Dobbs on PPD rates appears limited, it emphasizes the need for continued vigilance in monitoring maternal mental health outcomes, improving healthcare access, and addressing potential disparities in states with restricted abortion access in the post-*Dobbs* landscape.

### Ethics Approval and Consent to Participate

The raw data set on which this study is based is available through a commercial data licensing agreement with Kythera Labs. Kythera data have been expertly determined by Datavant’s Privacy Hub (Mirador) to comply with statistical de-identification required by HIPAA and associated regulations. The analysis of de-identified, publicly available data does not constitute human subjects research as defined by US Department of Health and Human Services 45 CFR §46.102 and does not require IRB review.

### Consent for Publication

As this study utilized de-identified data, patient consent to participate was not required.

### Availability of Data and Materials

The data that support the findings of this study are available from Kythera Labs under license for the current study and so are not publicly available. Data are available from Kythera Labs upon request and pursuant to a licensing agreement (https://www.kytheralabs.com/).)

### Disclosures

O.B. is Editor-in-Chief of the *Journal of Health Economics and Outcomes Research (JHEOR)*. A.E. is Managing Editor of *JHEOR*.

## Supplementary Material

Online Supplementary Material
