# Supplementary material for: The Effect of Dobbs v. Jackson Women’s Health Organization on Clinical Diagnosis of Postpartum Depression
Source: J Health Econ Outcomes Res. 2025 Feb 26;12(1):86–96. doi: 10.36469/001c.129633 (PMC11869945; doi:10.36469/001c.129633)
Supplement: Online Supplementary Material [file jheor_2025_12_1_129633_268395.pdf]

Journal of Health Economics and Outcomes Research

Online Supplementary Material

The Effect of *Dobbs v. Jackson Women's Health Organization* on Clinical Diagnosis of Postpartum Depression. *JHEOR*. 2025;12(1):86-96. doi:10.36469/jheor.2025.129633

Figure S1: Attrition Table for Study Sample

Table S1: ICD-10 Codes for Obstetrical, Maternal, and Lifestyle Risk Factors Identified

Figure S2: Variation of PPD Rates for All States Before and After *Dobbs*

Figure S3: PPD Rates of Trigger and Non-Trigger States Before *Dobbs*

Table S2: Parallel Trends Assumption Testing for the Pre-*Dobbs* Period Model

Figure S4: Heterogeneous Treatment Analysis Modeling Result by Stratifying Age Groups

Table S3: Heterogeneous Treatment Analysis Modeling Result Based on Callaway and Sant'Anna Estimator

Table S4: *Dobbs* Decision Effect for the Texas PPD Rates Prediction Model

Table S5: Patient Obstetrical Complications

This supplementary material has been provided by the authors to give readers additional information about their work.

This is an open-access article distributed under the terms of the Creative Commons Attribution 4.0 International License (CCBY-4.0). View this license's legal deed at http://creativecommons.org/licenses/by/4.0 and legal code at http://creativecommons.org/licenses/by/4.0/legalcode for more information.

Figure S1. Attrition Table for Study Sample

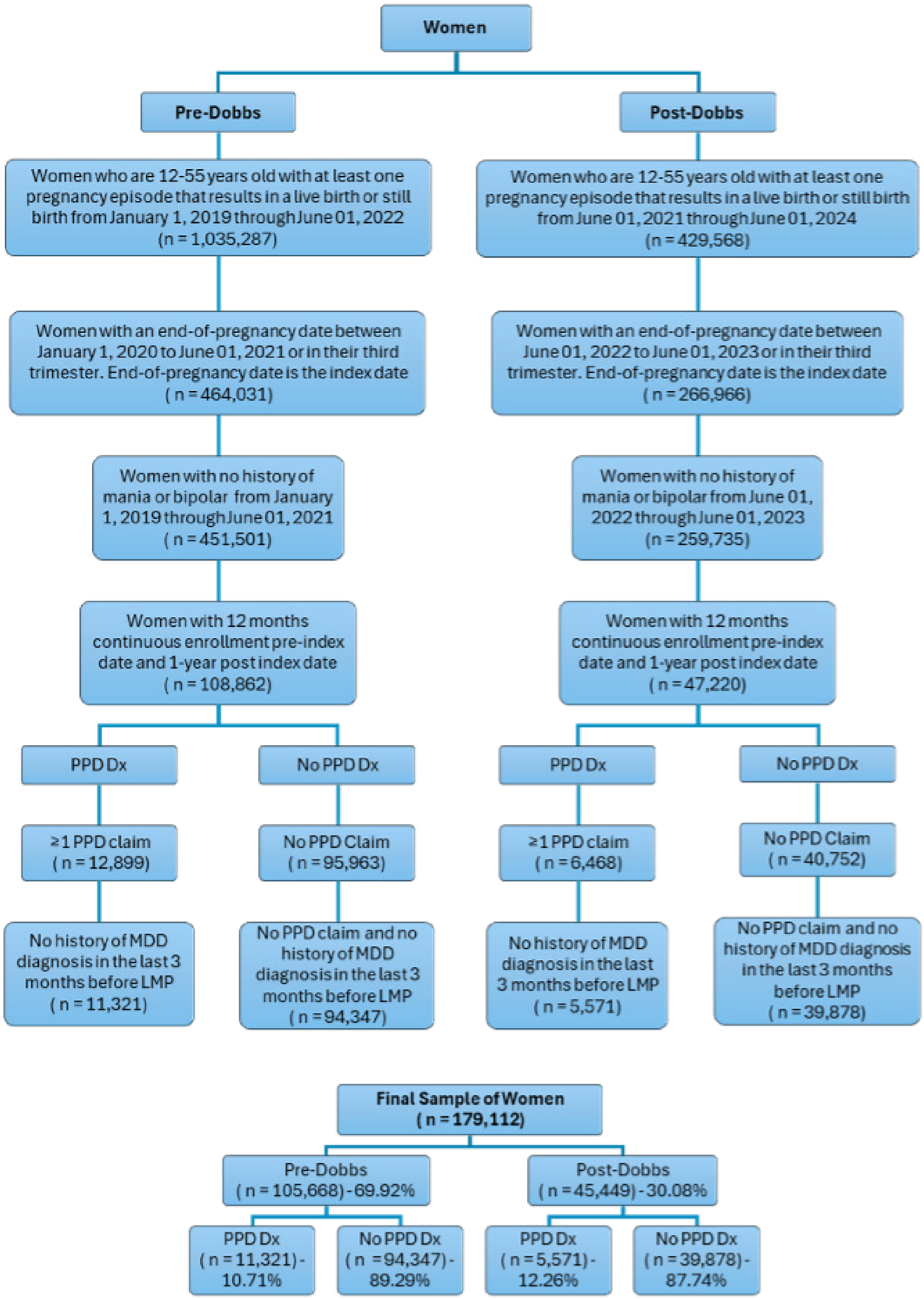

Abbreviations: Dx, diagnosis; LMP, last missed period; MDD, major depressive disorder; PPD, postpartum depression.

Table S1. ICD-10 Codes for Obstetrical, Maternal, and Lifestyle Risk Factors Identified

**Factors Identified**	**ICD-10 Codes**
Obstetrical complications	
Drug dependence	F19.20
High-risk pregnancy	O09.90, O09.899, O09.70
Severe perineal laceration	O703, O704
Postpartum hemorrhage	O72
Puerperal sepsis	O850
Infection of obstetric surgical wound	O860
Venous complication in puerperium	O870, O871, O873, O878, O879
Obstetric embolism	O88
Complication of anesthesia	O89
Complication of the puerperium	O90
Other maternal diseases	O98, O99
Cesarean section	O82
Multiple births	Z37.5, Z37.6, Z37.7
Preterm labor	O60
Abnormal findings	O28,O35,O36
Postpartum anemia	O90.81
Negative birth experience	Z87.59
Meconium passage	P03
Umbilical cord prolapse	O69.0
Prior history of abortion	Z33.2, N96, O02, O03*, O04*, O05*,O06*,O07*
Prior history of ectopic pregnancy	O09.10
Prior history of hydatidiform mole	O01.9
Other obstetric trauma	O71
Premature rupture of membranes	O42
Placental disorders	O43
Placenta previa	O44
Premature separation of the placenta	O45
Maternal comorbidities	
Preexisting hypertension	I10 obesity
Gestational hypertension	O13.3
Pre-eclampsia or eclampsia	O11, O13, O14, O15, O16
Gestational diabetes mellitus	O24
Pre-existing diabetes	E11.9
Lifestyle risk factors	
Vitamin D deficiency	E55.9
Obesity and overweight	E66
Sleep disorders	G47
Lack of physical exercise	Z72.3
Poor eating habits	Z72.4
Vitamin B6 deficiency	E53.1
Smoking	O99.330

Abbreviation: ICD-10, *International Classification of Diseases, Tenth Revision*.

Figure S2. Variation of PPD Rates for All States Before and After *Dobbs*

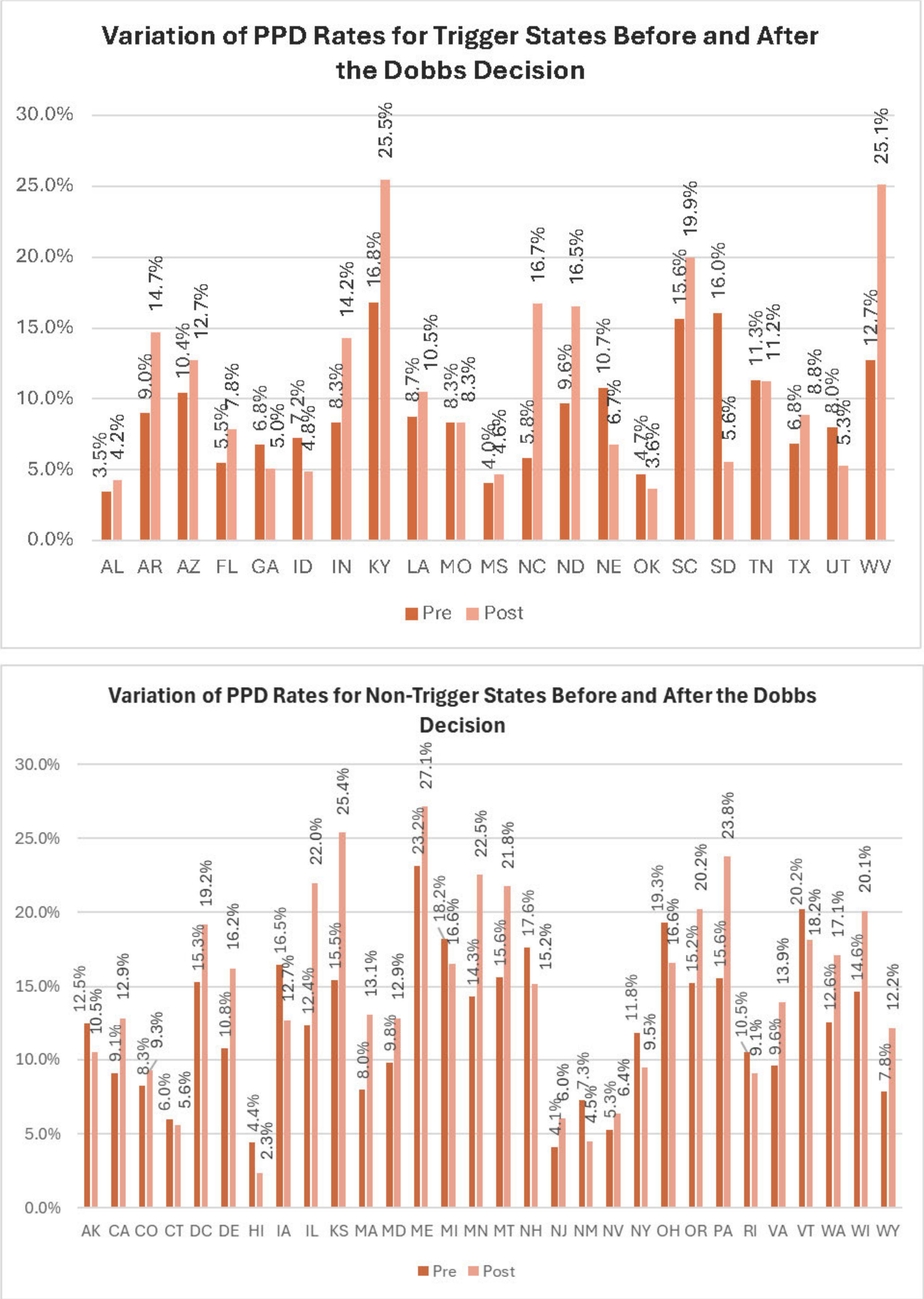

Abbreviation: PPD, postpartum depression.

Figure S3. Postpartum Depression Rates of Trigger and Non-Trigger States Before *Dobbs*

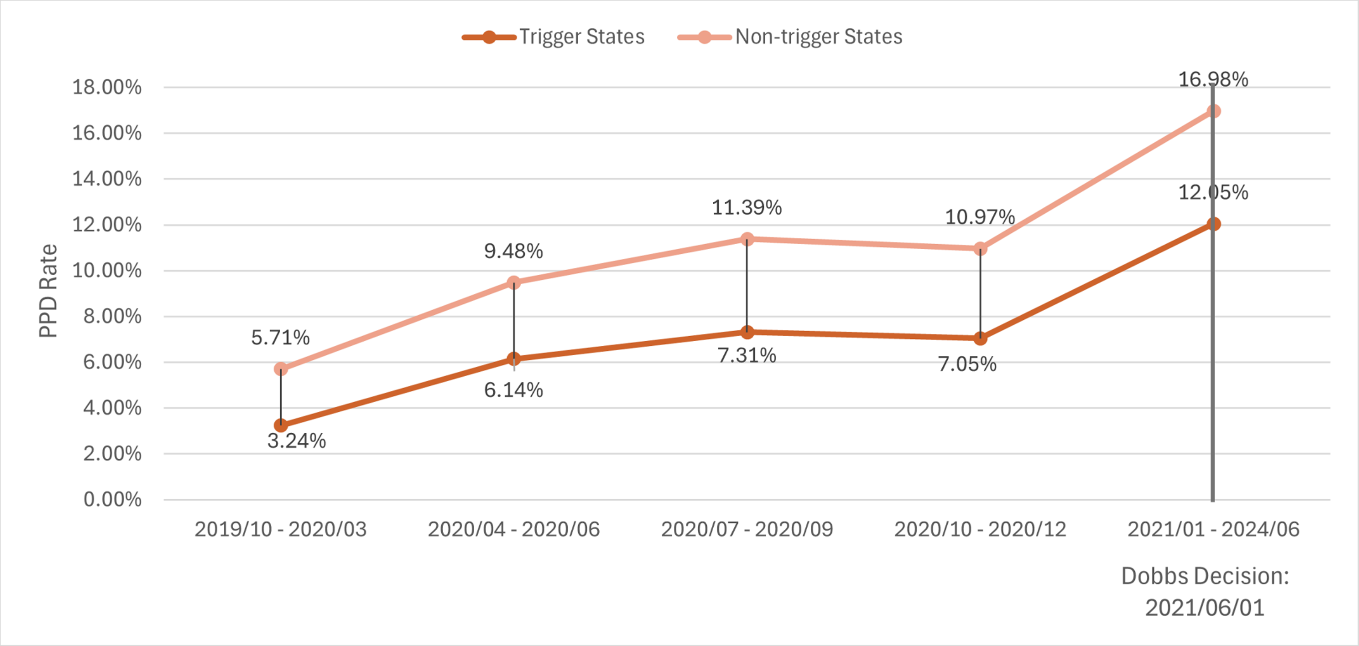

Table S2. Parallel Trends Assumption Testing for the Pre-*Dobbs* Period Model

Term	Estimate	Std. Error	t Value	Pr(>|t|)	Significance
(Intercept)	0.117969	0.002191	53.851	<2e-16	<.001
Pre-post	0.009353	0.002836	3.299	0.000972	<.001
Trigger1[a]	-0.039538	0.003095	-12.773	<2e-16	<.001
Pre-post:trigger1[b]	0.001614	0.004009	0.403	0.687299	
Residual standard error: 0.3049 on 96134 degrees of freedom					
Multiple R^2: 0.004255					
Adjusted R^2: 0.004223					
F statistic: 136.9 on 3 and 96134 degrees of freedom					
P value: <2.2e-16					

[a]Refers to the indicator variable (cases before 2020/07/09 = 0; cases after 2020/07/09 = 1).
[b]Refers to the indicator variable (trigger state = 1; non-trigger state = 0)

Figure S4. Heterogeneous Treatment Analysis Modeling Result by Stratifying Age Groups

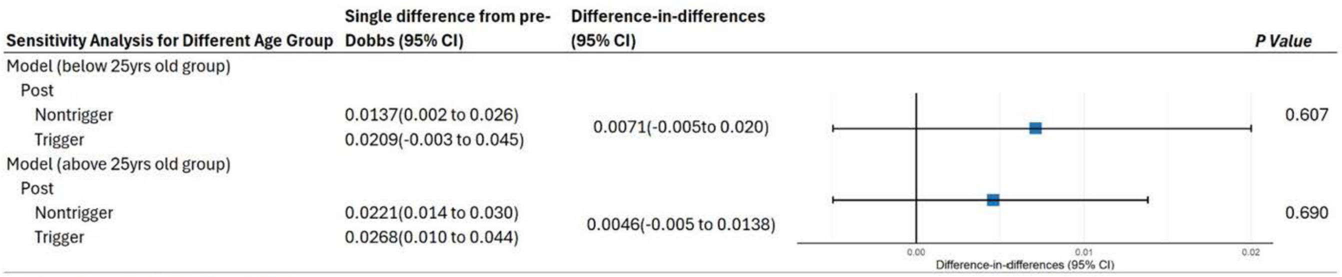

Table S3. Heterogeneous Treatment Analysis Modeling Result Based on Callaway and Sant'Anna Estimator

	**Study Estimate**
ATT(g,t)	0.0003
Std. error	0.0037
95% lower CI	-0.0072
95% upper CI	0.0072

Table S4. *Dobbs* Decision Effect for Texas Postpartum Depression Rates Prediction Model

	**Women 12-55**	
	**Unadjusted**	**Adjusted**
Post-*Dobbs*	0.013[a]	0.028[b]
Constant	0.126[b]	0.114[b]
	0.000472	0.005458
Residual std. error	0.258	0.2574
F statistic	6.541	10.73
P value	.01055	<.001[b]

[a]$P < .01$.
[b]$P < .001$.

Table S5. Patient Obstetrical Complications

	**Pre-*Dobbs* (N = 105 668)**					**Post-*Dobbs* (N = 45 359)**				
	**Trigger States (N = 48 775)**		**Non-Trigger States (N = 56 399)**		**Std. Diff.**	**Trigger States (N = 22 785)**		**Non-Trigger States (N = 22 574)**		**Std. Diff.**
	**N**	**%**	**N**	**%**		**N**	**%**	**N**	**%**	
Obstetrical complications, n (%)										
Drug dependence	66	0.14	149	0.26	0.0281	34	0.15	35	0.16	0.0013
High-risk pregnancy	2305	4.73	4558	8.08	0.1345	1437	6.31	2599	11.51	0.1823
Severe perineal laceration	6	0.01	7	0.01	0.0000	1	0.00	1	0.00	0.0055
Postpartum hemorrhage	93	0.19	169	0.30	0.0217	66	0.29	64	0.28	0.0014
Puerperal sepsis	0	0.00	0	0.00		0	0.00	0	0.00	
Infection of obstetric surgical wound	9	0.02	8	0.01	0.0035	2	0.01	2	0.01	0.0000
Venous complication in puerperium	4	0.01	3	0.01	0.0036	0	0.00	3	0.01	0.0163
Obstetric embolism	56	0.11	80	0.14	0.0072	24	0.11	40	0.18	0.0190
Complication of anesthesia	4	0.01	4	0.01	0.0013	3	0.01	1	0.00	0.0093
Complication of the puerperium	175	0.36	284	0.50	0.0221	138	0.61	107	0.47	0.0176
Other maternal diseases	24 707	50.66	34 660	61.45	0.2167	12 847	56.38	15 112	66.94	0.2167
Cesarean section	149	0.31	250	0.44	0.0221	67	0.29	83	0.37	0.0126
Multiple births	1	0.00	1	0.00	0.0007	1	0.00	0	0.00	0.0094
Preterm labor	3022	6.20	3383	6.00	0.0087	1246	5.47	1106	4.90	0.0253
Abnormal findings	16 827	34.50	24 160	42.84	0.1711	8 548	37.52	10 630	47.09	0.1936
Postpartum anemia	71	0.15	121	0.21	0.0165	50	0.22	47	0.21	0.0026
Negative birth experience	2085	4.27	4218	7.48	0.1341	1422	6.24	2469	10.94	0.1674
Meconium passage	18	0.04	36	0.06	0.0117	10	0.04	12	0.05	0.0041
Umbilical cord prolapse	6	0.01	5	0.01	0.0034	4	0.02	6	0.03	0.0060
History of abortion	1657	3.40	2510	4.45	0.0535	932	4.09	1061	4.70	0.0295
Prior history of ectopic pregnancy	24	0.05	47	0.08	0.0135	18	0.08	27	0.12	0.0128
Prior history of hydatidiform mole	6	0.01	5	0.01	0.0034	3	0.01	1	0.00	0.0093
Other obstetric trauma	211	0.43	291	0.52	0.0124	128	0.56	160	0.71	0.0182

Table S5. Patient Obstetrical Complications

	**Pre-*Dobbs* (N = 105 668)**					**Post-*Dobbs* (N = 45 359)**				
	**Trigger States (N = 48 775)**		**Non-Trigger States (N = 56 399)**		**Std. Diff.**	**Trigger States (N = 22 785)**		**Non-Trigger States (N = 22 574)**		**Std. Diff.**
	**N**	**%**	**N**	**%**		**N**	**%**	**N**	**%**	
Premature rupture of membranes	965	1.98	1218	2.16	0.0120	500	2.19	503	2.23	0.0017
Placental disorders	936	1.92	2060	3.65	0.1040	583	2.56	875	3.88	0.0739
Placenta previa	1410	2.89	2397	4.25	0.0726	644	2.83	892	3.95	0.0616
Premature separation of the placenta	125	0.26	223	0.40	0.0237	52	0.23	81	0.36	0.0239
Any obstetrical complications	32 219	66.06	43 628	77.36	0.2521	15 832	69.48	18 150	80.40	0.2522
Maternal comorbidities, n (%)										
Preexisting hypertension	1578	3.24	1998	3.54	0.02	964	4.23	1025	4.54	0.02
Gestational hypertension	1704	3.49	2056	3.65	0.01	917	4.02	887	3.93	0.01
Pre-eclampsia or eclampsia	4315	8.85	5258	9.32	0.02	2267	9.95	2318	10.27	0.01
Gestational diabetes mellitus	3821	7.83	6131	10.87	0.10	2056	9.02	2543	11.27	0.07
Preexisting diabetes	754	1.55	1107	1.96	0.03	422	1.85	472	2.09	0.02
Any maternal comorbidity	8006	16.41	11 053	19.60	0.08	4321	18.96	4750	21.04	0.05
Lifestyle risk factors, n (%)										
Vitamin D deficiency	898	1.84	1 241	2.20	0.02	680	2.98	840	3.72	0.04
Obese and overweight	5361	10.99	10 174	18.04	0.20	3199	14.04	4885	21.64	0.20
Sleep disorders	590	1.21	887	1.57	0.03	358	1.57	477	2.11	0.04
Lack of physical exercise	1	0.00	8	0.01	0.01	7	0.03	4	0.02	0.01
Poor eating habits	5	0.01	9	0.02	0.00	2	0.01	12	0.05	0.03
Vitamin B6 deficiency	0	0.00	3	0.01	0.01	0	0.00	3	0.01	0.02
Smoking	369	0.76	648	1.15	0.04	163	0.72	189	0.84	0.01
Any lifestyle risk factors	6626	13.58	11 942	21.17	0.20	4024	17.66	5768	25.55	0.19

Abbreviation: Std. Diff., standardized difference.
